# Incontinentia pigmenti: report on data from 2000 to 2013

**DOI:** 10.1186/1750-1172-9-93

**Published:** 2014-06-24

**Authors:** Francesca Fusco, Mariateresa Paciolla, Matilde Immacolata Conte, Alessandra Pescatore, Elio Esposito, Peppino Mirabelli, Maria Brigida Lioi, Matilde Valeria Ursini

**Affiliations:** 1 Institute of Genetics and Biophysics ‘Adriano Buzzati-Traverso’, IGB-CNR, Naples, Italy; 2 University of Basilicata, Potenza 85100, Italy; 3 Fondazione SDN IRCCS, Via E. Gianturco 113, 80143 Naples, Italy

**Keywords:** Incontinentia pigmenti, Genomic disorder, Neuroectodermal disorder, Molecular diagnosis, Registry, Database

## Abstract

We report here on the building-up of a database of information related to 386 cases of Incontinentia Pigmenti collected in a thirteen-year activity (2000–2013) at our centre of expertise. The database has been constructed on the basis of a continuous collection of patients (27.6/year), the majority diagnosed as sporadic cases (75.6%). This activity has generated a rich source of information for future research studies by integrating molecular/clinical data with scientific knowledge. We describe the content, architecture and future utility of this collection of data on IP to offer comprehensive anonymous information to the international scientific community.

## Introduction

Incontinentia pigmenti (IP; OMIM#308300) is a rare multisystemic genomic disorder with an estimated prevalence at birth of 0.7/100,000 [1]. IP is X-linked and usually lethal in males, and affecting the skin, but also other neuroectodermal tissues, in females. The skin lesions are the first clinical manifestations that appear, starting in the neonatal period with a vesiculobullous eruption (Stage I) and following a three stage evolution varying in duration from months to years, namely a verrucous stage (Stage II), a hyperpigmented stage (Stage III), and finally a hypopigmented stage (Stage IV) usually continuing throughout life [2,3]. Such skin defects, that follows Blaschko lines, are always present in IP and are therefore considered the main diagnostic criteria for IP according to Landy and Donnai (1993) [2]. The severity of the disease is related to the presence of neurological and/or ocular impairment [4]. Overall, the prevalence of functional Central Nervous System (CNS) manifestations is approximately 30% [5,6] ranging from a single-seizure episode to severe motor and intellectual disability [7]. Ophthalmologic abnormalities are present in approximately 20%–37% of IP patients [5,6,8]. IP is due to a mutation of the X-linked *IKBKG/NEMO* gene (*Inhibitor of Kappa polypeptide gene enhancer in B-cells, Kinase Gamma/Nuclear Factor* κ*B, Essential Modulator,* GenBank NM_003639.3, OMIM#300248). Most cases have a recurrent deletion (*IKBKGdel or NEMOdel4-10*), removing exons 4–10 of the *IKBKG/NEMO* gene. Non recurrent genomic rearrangements in the IP *locus* and point mutations in the *IKBKG/NEMO* coding region have also been reported [9-11]. *IKBKG*/*NEMO* encodes for NEMO/IKKγ a regulatory subunit of the Inhibitor of the kappaB (IκB) Kinase (IKK) complex required for the canonical NF-κB pathway activation involved in many fundamental physiological and pathological functions [12,13]. Most IP female patients present with a skewed X-inactivation. The X-chromosome linked *IKBKG/NEMO* mutation causes an unbalanced X-inactivation in female IP patients [14], as in other X-linked diseases [15,16], because the absence of the NEMO/IKKγ protein makes the IP cells more sensitive to apoptosis [9]. In males, the extensive apoptosis is responsible for their early fetal lethality [17]. Occasionally, male patients with IP have been reported. They have shown the characteristic skin lesions observed in females and presented a postzygotic mosaicism for the *IKBKG/NEMO* exons4-10 gene deletion [18]. IP has also been diagnosed in males with a 47,XXY karyotype (Klinefelter syndrome) [19]. The large heterogeneity of defects, the severe clinical presentations, and the wide spectrum of *IKBKG/NEMO* alterations [7,11,14,20] makes the selection of homogeneous groups of patients difficult, precluding any therapeutic approaches. Indeed, despite the considerable progress that has been made in detailing the basic pathology of the IP disorder, the gap between research and clinical care has remained wide. Moreover, the paucity of patients collected at each single diagnostic centre makes an overall epidemiological report difficult. The integration of scattered resources may be crucial for the success of future scientific accomplishments.

## Methods

**Figure 1:**
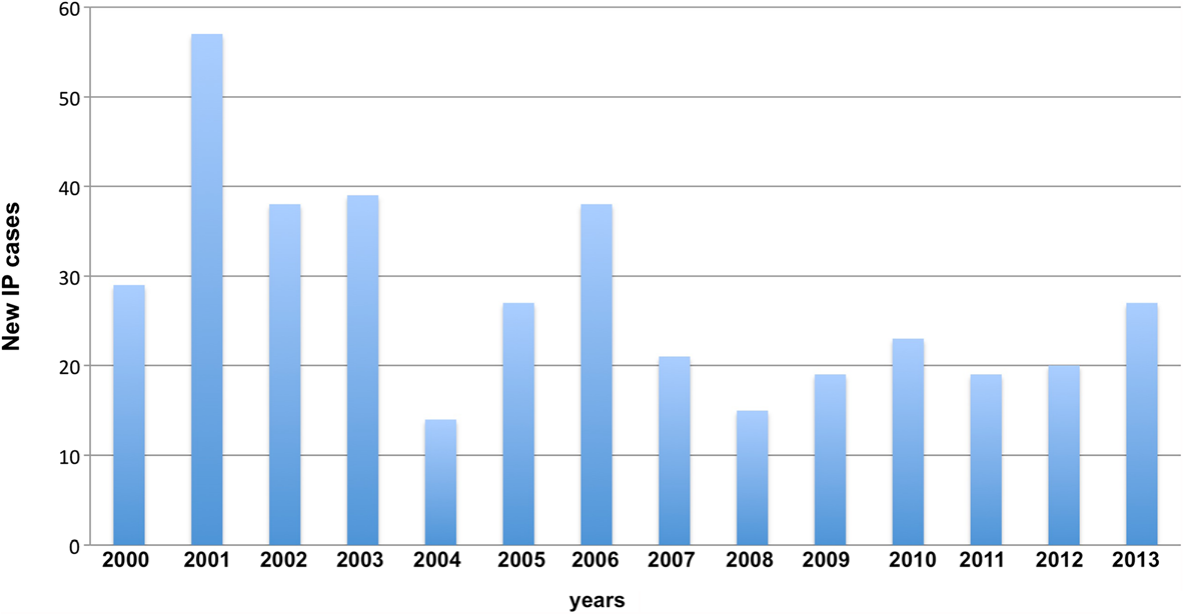
Annual distribution of the IP samples that have been received by the IGB centre for molecular diagnosis.

Here, we report the setting up of a central data repository relating to a cohort of IP patients, the data having been collected in a 13-year-long experience (2000–2013) at our Italian centre of expertise for the molecular diagnosis of IP [21]. The IP patients included in our study have been selected on the basis of the Landy and Donnai (1993) [2] diagnostic criteria, and they also meet the most recently updated IP criteria [22]. We have constructed the first platform for the integration of molecular and clinical data on IP patients. Our sample comprises 386 patients (261 from Italian, 105 from European and 20 from non-European clinical centers), with an annual average of 27.6 new cases of IP diagnosed per year (Figure 1).

All the clinical information has been obtained for each patient through their completion of a clinical IP questionnaire developed by the Incontinentia Pigmenti International Foundation (IPIF, [http://www.ipif.org/ip_consortium.html], further extended by the France Incontinentia Pigmenti association (FIP, [http://incontinentia-pigmenti.fr/]) and by the Italian ASSociation of Incontinentia Pigmenti (IPASSI, [http://www.incontinentiapigmenti.it/]). A clinical IP questionnaire is available upon request from these organizations. We have integrated the clinical data with molecular diagnosis results for the *IKBKG/NEMO* alteration, by way of a well-standardized protocol [10,14,23]. The technical development of the register has involved significant preparatory work consisting in the building up of an in-house electronic database which is comprehensive and permissive, and which has a flexible structure able to register in an anonymous form the pool data from the patients. We have assigned one record to each IP sample, registered with a pseudonymous code. Each record has three domains: the pedigree, the clinical and the genetic domain. The Web domain will be available at link [http://www.igb.cnr.it/ipgb]. The data are not accessible to everyone but only to authorized users through the use of a protected password. A data-mining interface has been developed to ensure maximum flexibility so that users can perform any search they want using the “search” button placed in the homepage after the “log in”. It is possible to perform multiple searches at once. To make the database permissive and flexible the first page contains only the three domains set.

## Results

The pedigree and clinical information are available for 308 IP cases, while the genetic data are available for 193 samples, respectively (Table 1). The pedigree domain contains more detailed entries accommodating the family data, for example the presence of an IP mother, sister, or grandmother, indicating the inheritance of the disease. We have registered 233 sporadic cases (75.6%) and 72 familial cases (23.4%) in our IP cohort.

**Table 1:** IP data registry

	**Number of cases**	**Percentage of cases**
***Database information***	386 records	
IP female samples	349	90.4
IP male samples	37	9.6
***Pedigree domain***	308 available	
Sporadic cases	233	75.6
Familial cases	72	23.4
Clinical domain*	308 available	
Skin defects	308	100
CNS defects	97	31.5
Ophthalmologic defects	94	30.5
Teeth defects	134	43.5
Hair defects	82	26.6
Fingernail defects	45	14.6
Developmental evolution	30	9.7
***Genetic domain***	193 available	
*NEMOdel4-10*	145	75.1
*IKBKG/NEMO* point mutation	32	22.1
IP *locus* rearrangement	7	3.6
No known alteration found	9	4.7

*IP patients can have more than one defect affecting different organ systems.

The clinical domain consists of seven clinical items, one for each aspect of the phenotype presentation: “*Skin* defects”, “*CNS* defects”, “*Ophthalmologic* defects”, “*Teeth* defects”, “*Hair* defects”, “*Fingernail* defects” and “*Developmental* evolution”. A drop-down menu has been assigned to each item that, in most cases, contains details about all the specific alterations affecting the tissue, and, in addition, an open space for the annotation of novel clinical features. For example, the item “*Skin* defects*”* contains a drop-down menu indicating the stage of the IP skin abnormality, the age of onset, the type of alteration, and the region of the body in which the alteration is present. We report that the most frequent first symptoms leading to diagnosis, typically skin alterations (Stage I), appear before the first year in 99% of cases. The second stage and the third stage are reported within the first year in 96.6% and in 82.8% of cases, respectively. After this date the fourth stage is generally present (Table 2). The specific frequency of each IP specific neuroectodermal defect observed in our cohort is shown in Table 1. CNS abnormalities were present in 31.5% (Table 1). In 17 cases, these were diagnosed by magnetic resonance imaging (Table 3).

**Table 2:** IP skin clinical data

		**Skin alteration age of onset**		
***Skin defects***	**IP cases**	**<1° month**	**1° month-1° year**	**>1° year**
Stage I	183	160(87.4%)	21(11.5%)	2(1.1%)
Stage II	90	38(42.2%)	49(54.4%)	3(3.3%)
Stage III	87	18(27.6%)	58(55.2%)	11(17.1%)
Stage IV	81	0	0	81(100%)

**Table 3:** IP clinical data

**Type of defect**	Number of cases**	**Percentage of cases**
***CNS defects***	***97***	
Seizures	39	40.2
Mental retardation	29	29.9
Spastic paresis	16	16.5
Cerebral atrophy	13	13.4
Microcephaly	11	11.3
Hydrocephaly	5	5.1
Ischemic strokes*	5	5.1
White matter alterations*	4	4.1
Arachnoid cysts*	3	3.1
Cortico-subcortical atrophy*	3	3.1
Brain morphological alterations*	2	2.1
***Teeth defects***	***134***	
Delayed primary dentition	46	34.3
Cone/peg shaped teeth	30	22.3
Delayed permanent dentition	30	22.3
Teeth dystrophy	23	17.2
Impactions	23	17.2
***Ophthalmologic defects***	***94***	
Vision defects	16	17
Retinopathy	15	15.9
Retinal detachment	8	8.5
Microphthalmia	6	6.4
Retinal neuropathy	6	6.4
Retinal vascular visorders	5	5.3
***Hair defects***	***82***	
Alopecia	8	9.7
Hypertrichosis	3	3.6
***Fingernail defects***	***45***	
Nail dystrophy	29	64
***Developmental evolution***	***30***	
Recurrent infections	36	11.7
Syndactyly of fingers or toes	4	1.3

*Data obtained by Magnetic Resonance Imaging analysis in 17 IP cases.

**IP patients can have more than one defect affecting different organ systems.

The genetic domain of the database contains four items: “*NEMOdel4-10*”, “*IKBKG/NEMO* point mutation”, “IP *locus* rearrangement”, and “No known alteration found” (Table 1). The mutation names comply with the accepted guidelines proposed by the Human Genome Variation Society [http://www.hgvs.org/mutnomen] [24]. The genetic domain reveals that 75.1% of patients have the *NEMOdel4-10* deletion, 22.1% have an *IKBKG/NEMO* point mutation, 3.6% have an extended deletion in the *IKBKG/NEMO locus*, and 4.7% have no known alteration in the IP *locus* (Table 1).

Finally, in each domain (pedigree, clinical, and genetic) an additional item, named “*Supplementary Information*”, records in the database any supplementary data from the patient and his/her family when these are available (examples: presence of miscarriages; presence of nonpathogenic alterations in IP *locus*[25] such as deletions or point mutations in the *NEMO* pseudogene).

## Discussion

The building up of this database represents the first detailed integrated clinical/molecular diagnostic platform on IP patients, the largest collection of an IP cohort in Italy and to the best of our knowledge the first presented to the scientific community worldwide. Thus, this phenotype and genotype database related to IP acts as a unique attempt to improve patient care and healthcare planning since it collects together information that would otherwise be scattered. Finally, we strongly believe that the use of the database is a powerful tool to facilitate the selection of biological samples and/or the enrolment of patients for the organization of appropriate clinical trials. Physicians wishing to include patients in the IP database may contact the Authors: Francesca Fusco and/or Matilde Valeria Ursini by email (incontinentia.pigmenti@igb.cnr.it).

## Abbreviations

IP: Incontinentia Pigmenti; OMIM: Online Mendelian Inheritance in Man; CNS: Central Nervous System; IKBKG: Inhibitor of Kappa polypeptide gene enhancer in B-cells, Kinase gamma; NEMO: Nuclear factor κB Essential MOdulator; IKKγ: Inhibitor of Kappa Kinase gamma; IκB: Inhibitor of NF-κB; IKK: IκB Kinase; NF-κB: Nuclear Factor-κB; IPIF: Incontinentia Pigmenti International Foundation; FIP: France Incontinentia Pigmenti association; IPASSI: Italian Incontinentia Pigmenti ASSociation.

## Competing interests

The authors declare that they have no competing interests.

## Authors’ contributions

FF: drafted the main part and created the final version of the manuscript, and designed the clinical and scientific content of the database, MP: implemented the content of the database, MIC: contributed to the content of the database, AP: contributed to the content of the database, EE: contributed to the content of the database, PM: contributed to the content of the database, MBL: participated in the design of the database- architecture and the data security concept of the registry, MVU: contributed to the content design of the database, and reviewed and revised the manuscript. All authors read and approved the final manuscript.
